# Parents’ perception of their children’s process of reintegration after childhood cancer treatment

**DOI:** 10.1371/journal.pone.0239967

**Published:** 2020-10-01

**Authors:** Laura Inhestern, Mona L. Peikert, Konstantin A. Krauth, Gabriele Escherich, Stefan Rutkowski, Daniela Kandels, Corinna Bergelt

**Affiliations:** 1 Department of Medical Psychology, University Medical Center Hamburg-Eppendorf, Hamburg, Germany; 2 Department of Pediatrics, Pediatric Hematology & Oncology, Klinik Bad Oexen, Bad Oeynhausen, Germany; 3 Department of Pediatric Hematology and Oncology, University Medical Center Hamburg-Eppendorf, Hamburg, Germany; 4 Swabian Children’s Cancer Center, University Hospital Augsburg, Augsburg, Germany; German Cancer Research Center (DKFZ), GERMANY

## Abstract

Our objective was to further the understanding of the process of reintegration of childhood cancer patients after treatment and to identify factors influencing that process. Using a qualitative approach, we conducted 49 interviews with parents (n = 29 mothers, n = 20 fathers) from 31 families with a child (<18 years) with leukemia or CNS tumor. Interviews were conducted about 16 to 24 months after the end of the treatment. We used a semi-structured interview guideline and analyzed the data using content analysis. Average age of pediatric cancer patients was 5.5 years at the time of diagnosis; mean time since diagnosis was 3.5 years. Parents reported immediate impact of the disease on their children. Reintegration had gone along with delayed nursery/school enrollment or social challenges. In most cases reintegration was organized with a gradual increase of attendance. Due to exhaustion by obligatory activities, reintegration in leisure time activities was demanding and parents reported a gradual increase of activity level for their children. Parents described several barriers and facilitators influencing the reintegration process into nursery/school and leisure time activities (structural support, social support, health status, intrapersonal aspects). Although many children reintegrate well, the process takes lots of effort from parents and children. Childhood cancer survivors and their families should be supported after the end of intensive treatment to facilitate reintegration.

## Introduction

During the past decades survival rates of childhood cancer patients have considerably increased to over 80% across all diagnoses [1]. Treatment, side effects and avoidance of infections are the primary priority in terms of cancer care during the acute phase of the disease [2]. Consequently, survival of a childhood cancer diagnosis requires major constraints during a vulnerable phase concerning the physical and emotional development of a child and can be associated with major adverse effects and long-term consequences [3–5]. During treatment children experience a disruption of their daily life and their age-specific development. They may drop out of nursery or school, may have physical impairments increasing the dependence on their parents and become aware of their physical vulnerability [6]. Childhood cancer survivors may experience social isolation, have fewer friends and may be less sociable [7, 8].

Although international guidelines recommend to identify and address psychosocial issues in survivorship [9–13], survivorship care mainly focuses on medical follow-up and identification and prevention of physical long-term consequences [14, 15]. After completion of treatment the shift from focusing on surviving the cancer disease to living with the consequences of the diagnoses and treatment has been described as a crucial phase for reintegration and adjustment [16]. Patients and caregivers describe the reentry into school as challenging [17]. Moreover, children may go through difficulties with regard to reintegration into social life [18].

Several factors can influence reintegration into nursery/school or social activities. Physical or cognitive late effects may cause difficulties in the reintegration to school [19]. Reintegration can be impeded by negative medium-term and long-term consequences of the disease and its treatment [20]. Treatment options depend on the type of cancer and the treatment protocol for the specific diagnosis. Additionally, symptom treatment and treatment for side effects of the cancer treatment are relevant in the comprehensive cancer care. Most frequent treatment options are surgery, chemotherapy, immunotherapy, radiation therapy or bone marrow/stem cell transplantation. Depending on the dose and the tolerability, side effects and long-term effects can differ [21]. Heart problems, low physical fitness, osteoporosis or rheumatological issues are reported long-term issues associated with cancer and its treatment [21]. Children after treatment for Medulloblastoma show cognitive impairments e.g. low perceptual organization or low processing speed factor [22]. Additionally, impairments such as poor balance, hearing impairments or neuropathy may influence the reintegration of affected children after cancer treatment [21]. Impairments and limitations due to childhood cancer and treatment may lead to performance limitations and, hence, lead to participation restrictions [21]. Individual functioning of the children are associated with reintegration [23]. Besides physical long-term effects, for teenagers changes in their appearance, e.g. due to hair loss, may result in difficulties concerning their self-confidence [24]. Additionally, family factors (e.g. parental functioning, family structures or coping skills) have been found to influence reintegration [16, 18]. Association between parental distress and child’s functioning indicate a close relationship between the family members’ condition [25]. A supportive social environment (e.g. teachers, peer group) and staying connected to school and classmates seem to facilitate reentry in school [2, 26–28].

In pediatric care, parents can be understood as the main representatives of their child. They play a major role to recognize their child’s needs and may initiate the use of support offers, where necessary. Consequently, parents are central in identifying possible barriers and they are key figures in the process of reintegration [29]. Hence, their experiences and point of view are essential for the development of psychosocial-support interventions.

Since reintegration during the first months and years has been claimed to be crucial for the long-term reintegration, a further insight of the situation of affected children is important. The aim of our study was to further the understanding of the process of reintegration in nursery/school and leisure time during the first years after the completion of treatment of childhood cancer survivors and to identify factors influencing that process. Results may provide a basis to develop more tailored health care and survivorship care of affected children after the completion of treatment.

## Methods

This interview study is part of a mixed-methods prospective study on children affected by leukemia or brain tumor diagnoses and their families in cooperation with the HIT-MED Registry, the study registry of the SIOP-LGG 2004 study, the COALL study and the Rehabilitation Clinic Bad Oexen [30]. Since CNS tumors and leukemia are the most frequent cancer diagnoses in childhood cancer patients, we focused on these two entities. We conducted a qualitative study using semi-structured interviews to gain information on relevant themes and factors from the parental perspective during their child’s cancer trajectory and reintegration process. Results on changes and reintegration of the parents are presented elsewhere [31].

The study was approved by the local ethics committee (Ethics Committee of the Medical Chamber of Hamburg, PV5277) and written informed consent was obtained from all study participants.

### Participants

Recruitment of participants followed two approaches. First, we invited families, who were participating in the quantitative study arm of our mixed-methods study. One hundred thirteen families were invited to participate in the interview study and received study information and informed consent for the interview study at the last measurement time point of the prospective study (approximately 16–24 months after the completion of intensive treatment).

In the second approach of recruitment, potentially eligible patients were identified by the HIT-Med Registry and the COALL study. Families were recruited during an aftercare appointment in the clinic. Interested families were invited for participation and received written information and consent forms. Unfortunately, the number of patients and families invited by the second approach is unclear.

Both, biological and social parents were eligible for participation. Parents were eligible if their child had received a CNS tumor or leukemia diagnosis, the patient was <18 years at time of diagnosis and parents had sufficient language skills to participate. Exclusion criteria were high physical and/or mental burden (assessed by health care providers/study registries at study entry) and/or cognitive limitations. Study participation was voluntary and written informed consent was obtained from all study participants. Parents from 35 families gave their signed consent for participation in the interview study. Interviews were conducted with 29 mothers and 20 fathers from 31 different families. Parents from four families dropped out (n = 3 no interview appointment could be arranged, n = 1 incomplete contact data).

We conducted consecutive sampling of the families that agreed to participate in the interview study until the research team established that data saturation was reached. Data saturation was reached when according to both coders no new themes occurred in the analyses.

### Sample characteristics

The mean age of interviewed parents was 42.6 years (SD = 7.1 years, Range 26–65 years) (Table 1).

**Table 1:** Characteristics of the participating parents (n = 49) and their children with cancer n = 31).

Characteristics	n
Parents (n = 49)	
Female	29
Age (years; M, Range)	42.6 (26–65)
Family status	
*Living with the partner/other parent*	43
*Single Parent*	5
School education	
*≤ 10 years*	15
*11–13 years*	34
Employment status	
*Full-time employment*	19
*Part-time employment*	23
*Not gainfully employed (Housewife/Househusband*, *Maternity leave*, *Seeking employment)*	7
Childhood cancer patients (n = 31)	
Female	12
Age at time of diagnosis (years; M, Range)	5.5 (0–17)
Time since first diagnosis (years; M, Range)	3.5 (1–15)
Cancer diagnosis	
*Leukemia*	22
*CNS tumor*	9
Current Cancer treatment	
*Maintenance treatment*	4
*No current treatment*	27
Supportive cancer care after intensive treatment	
*4-weeks inpatient rehabilitation program*	30
*Exercise/physical therapy*	16
*Occupational therapy*	8
*Speech/language training*	7

The children diagnosed with cancer were 5.5 years on average at the time of diagnosis (SD = 4.5 years, Range 0–17 years). 71% of the children were diagnosed with leukemia and 29% with a brain tumor. Mean time since initial diagnosis was about 3.5 years. All children but one had received chemotherapy. Surgery and radiation was each received by 26% of the children. At the time of the interview, 27 of 31 children had finished treatment, four children still received maintenance treatment. All but one children participated in a 4-week inpatient rehabilitation program (n = 28 family-oriented rehabilitation, n = 2 rehabilitation for adolescents). In Germany, the family-oriented rehabilitation program addresses patients with childhood cancer (≤15 years), their parents and healthy siblings. The program provides individual or group therapies and activities as required for all family members in multiprofessional therapeutic teams of physicians, clinical psychologists, social education workers and other professionals.

Parents reported that their children utilized outpatient exercise/physical therapy (52%), occupational therapy (26%), and speech/language training (23%) after intensive cancer treatment.

### Data collection

We developed a semi-structured interview guideline covering questions on the process from diagnosis and treatment to the time after completion of treatment. To explore the experiences of the parents individually in-depth questions were asked (e.g. *could you provide an example/a typical situation*? or *how did you feel about that*?). The guiding topics and example questions relevant for the analyses in this publication are presented in Table 2.

**Table 2:** Overview of the interview items^A^.

Central topic	Topics to be covered	Example questions	Possible in-depth questions
Course of disease	• Diagnosis • Progress, Symptoms • Treatment, Side effects • Health care use after completion of treatment • Health care recommendations by clinic or rehabilitation clinic	• Which cancer was diagnosed? • When did you receive the diagnosis? • Which treatment did your child receive?	• Could you provide an example/a typical situation? • How did you feel about that?
Reintegration of the ill child in nursery/school	• process of reintegration and changes in nursery/school • associated factors with nursery/school reintegration • current situation and satisfaction with situation	• How was the impact of the disease on school/nursery? • Which changes in school/nursery did your child experiences due to the disease until today? • What factors did influence the situation?	
Reintegration of the ill child in social life and leisure time activities	• changes in social life and leisure time activities from diagnoses up to today • barriers and facilitators for participating in social life and leisure time activities • current situation and satisfaction with situation	• Which changes in social life and leisure time did your child experience due to the disease until today? • What were barriers or facilitators for reintegration?	

^**A**^ Original questions were in German language. The questions were translated into English language for the publication.

All interviews were conducted by members of the research team (LI, MLP), who are researchers in the field of (pediatric) psychooncology, psychologists (PhD, M.Sc.) and experienced in qualitative research. At the beginning of each interview a short introduction (person, setting, study aim, data confidentiality) was given. The first interview was a pilot interview to test the guideline and, since no major changes were conducted, was included in our analyses. Most interviews were conducted by telephone, one interview was conducted in person due to organizational issues. Field notes were taken during and after the interviews. The interviews lasted 43 minutes on average (range 20–112 minutes).

### Data analysis

All interviews were recorded and transcribed verbatim based on the Simple transcription system [32] using f4transkript software (version 4.2; www.audiotranskription.de). Transcripts were not returned to the interviewees for feedback. The transcripts were analyzed with the qualitative content analysis based on the approach of Mayring [33] using the software MaxQDA [34]. Themes and categories were generated deductively based on the guiding questions from the interview guideline. Further categories were derived from the transcript data inductively. LI and MLP independently generated an initial overview of themes and codes based on three interview transcripts. After discussion a draft of a coding system was developed and systemized independently by LI and MLP based on further seven interviews. The code systems were consolidated and revised and a final coding guideline including category labels, category definitions and anchor samples was developed [see Table 3 and S1 Table]. Based on this final coding system all interviews were coded by MLP. To determine the validity of the coding system, about 40% (n = 20) of the transcripts were coded by MLP and LI independently. The percent agreement between both coders was 85% over all codes.

**Table 3:** Factors influencing reintegration and exemplary quotes from n = 49 interviews with parents of childhood cancer patients.

Influencing factors	Subthemes	Anchor examples
**Structural support**	*Continuity of education (home/hospital teaching)*	*Basically it started in the clinic*, *there were pleasant teachers teaching him [the ill son]*, *so that the hospital schooling worked out really well*. *In the end*, *we owe it to the clinic as well as to our local school that everything worked out that smoothly*.
	*Supportive facilities (e*.*g*. *classroom assistance)*	*Though she is able to follow lessons*, *you notice that she reaches her limitations*. *Now*, *with the school support assistance*, *there is somebody who takes care of her and sometimes initiates a break for her*, *so that she can lie down for a while*, *take a nap or just relax for some time*.
	*Social legal advice*	*They advised us especially on applications*. *Support offers we can use and which we are entitled to*. *And we used just that*, *since we didn’t know what else we could do*. *Thus it was really good/helpful that they also were present [in the clinic]*.
	*Family-oriented rehabilitation program*	*That was a rehabilitation program for the child*, *with the child*, *around the child*. *They picked them up*, *in which ever miserable condition they were*. *And they got them fit*. *(…) She was really weak due to the intensive treatment and then had to get back to something like daily routines*. *It was great*. *You cannot say anything else*.
**Health status**	*Risk of infections*	*If anything*, *I’d say*, *a cold or gastrointestinal infections*, *anything*, *occurred [among the children at the nursery]*, *they [staff from nursery] immediately called me*. *And then he [the son] just stayed at home*.
	*Limited physical energy*	*What always surprised me is that she fell asleep at school during lessons*. *(…)*, *she was really too exhausted*.
	*Long-term consequences*	*Well*, *he isn’t able to stand on his legs the whole day*. *There he is impaired*. *He can only walk short distances*. *Can’t do any sports or something like that*. *He can ride his bike*, *walk a little and swim a little*. *But he is impaired in moving his joints and we have to watch out for him not to break any bones*.
**Intrapersonal aspects**	*Attitude of the parents*	*It was difficult for him that his class went on a school trip just when he got back to school*. *And I wasn’t ready yet to let him go*.
		*Days of absence were extremely elevated*. *But I always said to myself “I don’t want him to catch a serious infection [at school]”*. *(…) I sometimes felt that coming and said “You rather stay at home and rest”*. *(…) This is something I wouldn’t have done before*.
	*Character of the child*	*(…) [She] is*, *I’d say*, *goal-oriented and motivated*, *not always*, *but on the whole*. *What changed due to the illness is*, *that she is very serious and she rarely meets friends*. *That she is very quiet and reticent*.
		*Well*, *she is just an open-minded character*, *easily approaches others and makes new friends*.
**Social network**	*Peer contact during treatment*	*During the first year*, *during the intensive treatment*, *when he wasn’t allowed to visit school*, *we celebrated his birthday on a sports field*. *(…) And almost everybody came*. *(…) And we always wrote stories*. *A letter to the class and sometimes they wrote letters*. *There was surprisingly much communication*.
	*Supportive peer group*	*Well*, *the children in class know about her disease*, *but they are really nice and considerate*.
	*Supportive staff in nursery/school*	*It worked out quite well and he didn’t need to repeat a year*. *He was able to follow the lessons*, *also because his teacher supported that*. *Since he did already have such a stroke of fate with the disease*, *they wanted that he could at least stay in his class*.

## Results

The interview data were organized in the following main categories: immediate impact of the disease and treatment, process of reintegration in school/nursery, process of reintegration in leisure time activities and factors associated with the reintegration process.

### Immediate impact of the disease and treatment

**Fig 1:**
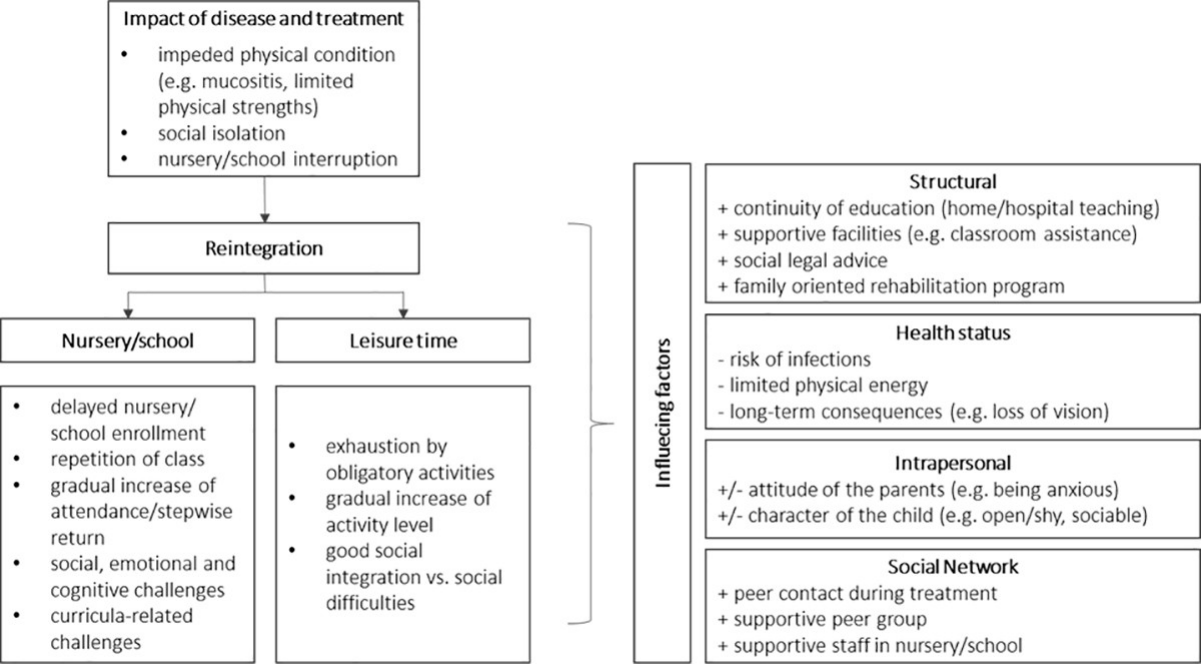
Reintegration of childhood cancer patients after treatment and influencing factors from the parental perspective (n = 49).

All parents (n = 49) mentioned a strong impact on the physical condition of the patient, e.g. mucositis, loss of appetite or swelling due to cortisone (Fig 1). Parents of eleven children also reported more severe physical consequences such as pneumonia or sinus thrombosis. Children had remarkable mood swings (ten of 31 children) and were impaired in their functioning (15 of 31 children). Moreover, parents of 26 children reported interruption of school or nursery of their children for several months, e.g. due to hospital stays or need of isolation. Hence, children were often socially isolated from their peer group and mainly had contact only with their parents or siblings and adults (15 of 31 children). To prevent their children from infections, parents used several strategies. Eleven parents described that they checked the health status of friends. Two parents used gloves and face masks when the ill child was playing with its siblings or friends. One father described his daughter being trapped in a “gilded cage”.

### Process of reintegration in nursery/school

According to the parents, at the time of the interviews 9 children visited nursery and 21 children visited school (n = 10 in primary school). Parents reported that reintegration into nursery or school had gone along with delayed school or nursery enrollment (eleven of 31 children) or had been associated with necessary repetitions of classes due to absence periods (three children). Most children were reintegrated to nursery or school shortly after completion of treatment and/or after an in-patient rehabilitation program after the completion of treatment. Parents described that the reentry in nursery or school was arranged with a gradual increase of attendance (eleven of 31 children). While some children attended nursery/school at the time of the interview to the same extent as before the diagnosis, others were still visiting school or nursery with reduced hours and many absent days.

*“He started full of optimism*. *He absorbs (…) life*, *he really does*, *new people and new school subjects*, *which he meets easily*. *And*, *he is always a little wistful*, *because of many infections going around*, *and vaccinations do not proceed*, *he is missing about one to three days a week*, *since he catches an infection*. *His immune system isn’t yet capable of that*.“

Parents reported that the reintegration was accompanied by curricula-related challenges (e.g. due to absence in school) (eleven children), social challenges (e.g. due to less peer contact during treatment) (twelve children), and emotional and cognitive challenges (e.g. difficulties to concentrate) (17 children) (Fig 1). However, most of the children encountered these challenges and adjusted well according to the parents.

### Process of reintegration in leisure time activities

Participation in activities apart from nursery or school was described as a challenge for many children. According to their parents, children were exhausted by the obligatory activities (nursery, school) early after completion of treatment (ten of 31 children). Parents of eight children reported a gradual increase in activity levels depending on the physical capacities of their children.

*“Well*, *what has changed because of the disease is*, *that she has become very serious*, *that she meets friends rarely*, *very rarely*, *that she*, *well*, *is just still very quiet and reticent*. *And*, *there was hardly any time for hobbies and little energy and strength*, *but it now slowly returns*.”

About 40% of the children started new hobbies (e.g. sports) after the end of treatment, while for other children it was difficult to follow their hobbies from before diagnosis due to physical limitations (five of 31 children). According to the parents, 17 of 31 children were well integrated, meeting friends after nursery or school. However, some children had less friends than before and had difficulties to integrate into the peer group (eleven of 31 children). Reasons were e.g. that they were insecure or had to re-learn how to act in a group of children.

*„Well*, *I think*, *when returning [to daycare] he was one child of many (…)*, *he was not the special child any more*. *And I think*, *that was hard for him*. *At the beginning he was aggressive*. *He tried to push in–that was difficult*.“

### Factors associated with the reintegration process

Parents reported several barriers and facilitators influencing the reintegration process into nursery/school and leisure time activities (Fig 1, Table 3).

#### Structural support

Parents reported aspects of structural support as key factors. Parents of ten children mentioned a continuity of education during treatment with home/hospital teaching as helpful for school reintegration. It allowed children to return to their former class and peer group. In school itself, six children had a classroom assistance and seven children had a disability compensation, which were proposed as facilitators for reintegration. Still, four parents mentioned that support for their children depended on parental commitment and persistency in dealing with nursery and school. According to four parents, social legal advice was a further supportive factor. In particular, the family-oriented rehabilitation program was reported by 32 of 49 parents to be important. In the rehabilitation families received a multiprofessional, 4-week inpatient program for the ill child and family members. It supported children in regaining physical strength and being in touch with other (affected) children.

#### Health status

Ten parents reported that during the first months after treatment, reintegration was impeded by the risk of infections. Parents avoided activities where the children possibly could get any infections due to the immunosuppression of their child. Moreover, limited energy due to the exhausting treatment phase and consequences was reported by 13 parents. Children were slowly regaining physical strength after the end of treatment. Still, it was physically and emotionally challenging for some children to follow their daily activities by the time of the interview. Twenty of 49 parents reported that their children suffered long-term physical consequences or cognitive consequences.

#### Intrapersonal aspects

Twenty-two of 49 parents considered personality traits of themselves as parents as an influencing factor for reintegration. While some parents emphasized the benefit of a shift of priorities towards a lower importance of school performance, some others reported that they had a tendency for overprotection. Thirty-two of 49 parents reported that the character of their children e.g. social skills or the child’s urging need to do things it had missed under treatment facilitated reintegration. Reentry into school was facilitated by intellectual abilities and willingness to learn.

#### Social network

Parents perceived social support to be essential after completion of treatment. In school or nursery, 28 of 49 parents perceived support from teachers, nursery nurses or heads of the institutions as facilitator for reintegration. Ten parents reported that support from local parents’ associations was essential during treatment but also after the end of treatment in terms of psychosocial support, useful tips and exchange with other families. Moreover, open-minded friends of the children who stayed in contact with the patients during the treatment, were reported to be a positive factor by 22 of 49 parents.

## Discussion

This study allows a further insight into the situation of childhood cancer survivors after completion of treatment. In our study, most children had reintegrated to nursery/school gradually and had, according to their parents, returned at the time of the interview. However, some children had frequently absences from school due to infections or exhaustion.

The experiences of the parents reinforce that children face specific challenges during reintegration after the end of treatment, which may not be captured by solely quantitatively analyzing quality of life levels [35]. Getting back to their new old “normal” life can lead to exhaustion and requires many resources from the children [17]. The time of reintegration poses new demands and difficulties after undergoing cancer treatment. Our results show that particularly participation in activities beyond the obligatory activities (nursery, school) requires time and energy, which many children cannot muster during the first years after treatment. At the same time, increased participation in e.g. organized sports may further social integration [36].

Our findings indicate that children with difficulties in reintegration could be identified early after the end of treatment. Offering adequate support may prevent long-term problems in childhood cancer survivors [4, 37]. Support offers should prepare patients and families for the process of reintegration and provide information with regard to physical and emotional consequences as well as guidance concerning medical follow-up and late effects [38].

Our findings on factors influencing reintegration highlight the importance of facilitators and impeding factors. Results indicate that the foundation for successful reintegration can already be established during treatment, e.g. in terms of enabling continuous education or staying in touch with peers. Parents worried about possible obstacles and therefore tending to overprotect their children may impede the reintegration process.

Particularly after the end of treatment, affected families and children experience declining social support and care [39, 40]. Hence, it can be important to communicate openly with peers and staff in school or nursery to mobilize social support networks.

In addition to social support, structural support was reported to be crucial as well. For example, the family-oriented rehabilitation program, a 4-week inpatient multidisciplinary program, can be essential for the reintegration after cancer treatment. Many families do not feel well supported after the end of treatment, but rather left alone [16]. Structured rehabilitation programs can help these families to prepare for reintegration with a multidisciplinary approach.

To facilitate children’s reintegration and ensure necessary support, independently from parents’ engagement and abilities, aftercare for childhood cancer survivors should routinely and systematically address psychosocial aspects. With regard to school reentry, programs to enhance collaboration between school, family and medical team have been developed and shown to be effective in terms of reintegration [25, 41]. However, such programs have not been routinely established in cancer care so far.

There are several limitations to consider when interpreting the study results. First, the qualitative approach comprises only experiences from the parental view. We did not assess the children’s perspective. Children’s perspective may provide additional information on relevant aspects for reintegration, which have not been mentioned by parents. Children, parents and other key figures (e.g. teachers) may experience e.g. the process of reintegration differently [29]. Since this study is a qualitative study, we did not survey certain issues or factors systematically. Hence, only aspects parents expressed and assumed to be important are included. Our results may rather provide relevant information for future studies on factors influencing reintegration during the first months and years after treatment. These factors could be assessed in a systematic manner and in long-term in future studies, which might allow further evidence on predictors for reintegration.

We also might underestimate the difficulties in the process of reintegration, since our sample included more parents of children with leukemia than with CNS tumors. It has been shown that CNS tumor patients suffer from severe limitations and difficulties, which may adversely affect reintegration [42]. Moreover, diagnoses such as sarcomas or other solid tumors, which can imply complex late effects, were not included in our sample. We cannot conclude anything on the representativeness of our sample with regard to demographic data of the families. Since our study sample mainly consists of highly educated parents and parents in a partnership, we might have missed the experiences of reintegration from families with lower education or from lower socio-economic background, with migration background or single parents, and hence, we cannot generalize our findings.

Our results allow new insights into the situation of childhood cancer survivors after end of treatment. It can be shown, that rigorous planning on health care and support offers during the early phase of survivorship is important for affected families. Cancer aftercare should focus on supportive needs during the process of reintegration. Patients seem to benefit from stepwise reintegration into nursery/school and supportive care offers such as a multidisciplinary rehabilitation programs. Besides structural support, particularly continuity of education and peer contact seem to be key factors in facilitating reintegration. Our findings may be used to design and evaluate interventions to support affected children and their families in particular after the end of treatment, e.g. a multidisciplinary approach with several modules which can be tailored according to individual needs.

## Supporting information

S1 Checklist (PDF)

S1 Table Example of coding guideline for the reintegration in school/nursery. (DOCX)
