# Supplementary material for: Parents’ perception of their children’s process of reintegration after childhood cancer treatment
Source: PLoS One. 2020 Oct 1;15(10):e0239967. doi: 10.1371/journal.pone.0239967 (PMC7529258; doi:10.1371/journal.pone.0239967)
Supplement: S1 Table — (DOCX) [file pone.0239967.s002.docx]

S1 Table

S1 Table. Example of coding guideline for the reintegration in school/nursery^A^

| **labels** | **category definitions** |
| --- | --- |
| **School/Nursery** |  |
| Process of reintegration to education |  |
| Procedure  Gradual increase  Repetition  Delayed enrollment | Difficulties/facilitations facing while reintegration; adaptations need to be made |
| Curricula-related challenges | Difficulties occurring due to missed tuition |
| Social challenges | Reactions/Interaction/Acceptance/Rejection by class mates after returning to school/nursery/repetition of grade |
| Emotionally/Cognitive challenges | Feelings and thoughts; any cognitive restriction caused by disease/treatment; consequences |
| Influencing factors on reintegration |  |
| Continuity of education | Attending classes offered at hospital/home |
| Compensatory measures | Compensatory measures (Disability compensation) that apply to patient due to disease/treatment |
| Classroom assistance | Support in school to compensate for physical or psychosocial impairments |
| Rehabilitation measures | 4-week inpatient rehabilitation program, improving cognitive abilities, mobility, social skills |
| Risk of infections | Actions taken to avoid any infection due to immunosuppression of the patient |
| Physical limitations | Physical/cognitive/emotional consequences |
| Parents’ attitude | Parents’ attitude towards school/nursery and parents’ resulting behaviour |
| Child’s character | Child’s attitude/character towards school/nursery and its resulting behaviour |
| Support by School/Teacher | Support offered by teachers; difficulties/ obstacles caused by teachers |
| Social support by extended family/friends | Support/No support by extended family/friends |
| Peer-group | Supportive or not supportive class mates and friends |

**^A^** Original labels and definitions were in German language. They were translated into English language for the publication.
